# Mechanism-Informed Health Representation and Causal Stage-Aware Remaining Useful Life Prediction for Rolling Bearings

**DOI:** 10.3390/s26206373

**Published:** 2026-10-09

**Authors:** Xiange Tian, Hongtao Yin, Siyu Chen, Zhao Dong, Haiyang Xu

**Affiliations:** 1 School of Electrical and Information Engineering, Jiangsu University, Zhenjiang 212013, China; tianxiange@ujs.edu.cn (X.T.); 2212407043@stmail.ujs.edu.cn (H.Y.); 2222507026@stmail.ujs.edu.cn (S.C.); 2212507086@stmail.ujs.edu.cn (Z.D.); 2 Institute of Ocean Technology, Ocean College, Zhejiang University, Zhoushan 316021, China

**Keywords:** bearing remaining useful life, health indicator, first prediction time, stage-aware learning, long short-term memory network

## Abstract

Accurate bearing prognostics require health representations that distinguish persistent degradation from transient vibration responses. This study develops a sequential framework comprising a mechanism-informed gated health indicator (MIG-HI), causal evidence-driven transition identification (CETI) and a stage-conditioned dual-branch long short-term memory network (SCDB-LSTM). MIG-HI groups multidomain vibration descriptors into structural, energy, impulsiveness, fault mechanism, and cross-channel evidence and regulates impulsive contributions using mechanism and channel support. CETI combines level, trend and persistence evidence to establish a formal first prediction time (FPT). This reference links an offline degradation-aligned normalized prognostic target with a causally updated stage coefficient. SCDB-LSTM combines branch-specific representations through a shared temporal encoder and conditions regression on the stage coefficient. The evaluation described here includes condition-specific prediction experiments under three PHM 2012 operating conditions and detailed health-indicator analyses on five Condition 1 bearings. MIG-HI achieves the highest trendability among the compared indicators (0.4967), although it does not dominate monotonicity or prognosability. The target is dimensionless and does not directly estimate remaining operating time. Cross-condition transfer, disturbance-specific robustness and calibrated predictive uncertainty require further validation.

## 1. Introduction

Rolling bearings are essential load-carrying components in rotating machinery, and their progressive degradation can reduce equipment availability, increase maintenance costs and eventually lead to unplanned shutdowns. RUL prediction estimates the remaining operating time or relative service life available before a predefined failure endpoint, thereby providing quantitative support for condition-based maintenance [1]. Existing prognostic approaches are commonly classified as model-based, data-driven and hybrid or knowledge-informed methods. Model-based methods provide interpretable descriptions of degradation but require sufficiently accurate physical or stochastic models, which are difficult to establish for bearings with complex failure mechanisms and heterogeneous degradation trajectories. Data-driven methods reduce this modeling requirement by learning degradation patterns directly from condition-monitoring data, but their reliability depends strongly on whether the observations can be transformed into a stable and prognostically meaningful representation. These complementary limitations motivate mechanism-informed data-driven prognostics, in which partial domain knowledge is used to organize degradation evidence without requiring a complete physical failure model [2].

Within data-driven bearing prognostics, deep learning has become a common approach for mapping condition-monitoring observations to RUL. Convolutional and temporal convolutional architectures have been employed to capture local and multiscale degradation patterns, recurrent networks to model sequential evolution, and graph- or attention-based models to characterize long-range and cross-observation dependencies [3,4,5,6]. Together, these developments have strengthened the capacity of prognostic models to learn nonlinear relationships from high-dimensional condition-monitoring data. However, increased model expressiveness does not necessarily produce a stable or interpretable representation of bearing health. Because the internal features of many end-to-end models are learned under supervision from the final RUL objective rather than explicit health-state constraints, they may encode operating fluctuations or transient disturbances alongside degradation-related information and therefore may not consistently reflect progressive degradation. This limitation is particularly relevant during incipient degradation, when weak fault-related changes coexist with background noise and isolated vibration impacts. Consequently, the quality of the health representation remains a prerequisite for reliable transition identification and subsequent RUL regression.

Health indicators (HIs) provide an explicit intermediate representation between vibration measurements and RUL prediction. Xu et al. [7] developed an unsupervised attention-based HI by fusing similarity measures between learned representations of healthy and damaged states. Guo et al. [8] constructed a nonlinear HI from multidomain vibration features. Yang et al. [9] integrated data-driven representations with degradation knowledge across the bearing life cycle, whereas Yin et al. [10] embedded physical knowledge into degradation-trajectory modeling. These studies have improved the nonlinear representation or physical consistency of bearing health evolution. However, the reliability of impulse-sensitive evidence under transient disturbances remains insufficiently addressed. Strong impulsive responses may arise from either localized bearing defects or isolated disturbances and should therefore be evaluated together with fault-frequency activity and cross-channel consistency. Otherwise, fixed fusion may cause premature HI excursions or early saturation. These considerations motivate an adaptive mechanism that regulates impulsive contributions using complementary mechanism-related support.

A reliable health trajectory is also required to identify the transition from nominal operation to predictable degradation. Whereas Wang and Xiang [11] modeled bearing degradation directly for RUL estimation using a gradient-optimized exponential process, subsequent studies have increasingly incorporated explicit transition information into prognostic modeling. Li et al. [12] adaptively detected a degradation-stage transition point before implicit Kalman filtering-based RUL estimation, whereas Li and Wang [13] constructed an HI intended to support both FPT detection and RUL prediction. Two-stage Bayesian and piecewise transfer-learning frameworks have also been developed to organize RUL estimation over different degradation intervals [14,15]. In addition, Guo et al. [8] used a 3σ criterion to determine FPT before nonlinear Wiener-process prognosis. These studies demonstrate the value of transition-aware prognosis, but the identified transition is used mainly to initiate degradation modeling, delimit the prediction interval, or partition life-cycle samples. Its simultaneous use in degradation-aware RUL supervision and causal stage representation remains less explored. Moreover, a level threshold may respond to isolated HI excursions, whereas a short-term trend does not necessarily indicate a persistent state transition. This motivates a causal strategy that jointly evaluates health-level deviation, trailing trend, and temporal persistence using only current and historical observations.

During degradation progression, the compact HI and multidomain vibration descriptors provide complementary prognostic information. Li et al. [16] incorporated risk assessment and a degradation-state coefficient into bearing RUL prediction. Bai et al. [17] developed a two-phase deep neural network for simultaneous health monitoring and prediction, whereas Qiu et al. [18] employed a piecewise temporal convolutional network for bearing RUL estimation. Gao et al. [19] combined adaptive stage division with long-term temporal attention. Wang et al. [20] proposed an adaptive staged RUL prediction framework. These studies demonstrate the value of distinguishing degradation stages through state weighting, life-cycle partitioning, or piecewise prediction. However, the combined problem of preserving the distinct representations of a compact HI and multidomain descriptors while using an FPT-referenced continuous stage variable to condition a unified causal temporal predictor remains less explored. Direct input concatenation may obscure the different prognostic roles of these information sources, while uniform treatment of all observations may overemphasize densely represented life-cycle regions. These considerations motivate a stage-conditioned architecture with branch-specific representations and bearing-stage-balanced learning.

Taken together, existing studies have established useful links among health representation, transition identification and stage-aware RUL prediction. However, these connections are generally developed for specific pairs of tasks, leaving the propagation of degradation information across the complete prognostic process insufficiently coordinated. In particular, mechanism-supported health evidence is not consistently propagated to causal transition confirmation, and the confirmed transition is not systematically reflected in both degradation-aware RUL supervision and causal stage representation. Meanwhile, the distinct roles of the compact HI and multidomain vibration features may be obscured during temporal modeling. These limitations indicate the need for an explicit information flow that coordinates health representation, transition identification, target construction, stage representation and RUL prediction.

To address these interdependent limitations, this study develops a sequentially coordinated framework comprising MIG-HI, CETI and SCDB-LSTM. MIG-HI constructs a mechanism-supported health trajectory by regulating impulsive evidence according to fault-frequency support and cross-channel consistency. Based on this trajectory, CETI jointly evaluates health-level deviation, trailing trend and temporal persistence to identify the formal FPT, which guides the construction of the degradation-aware normalized RUL label and the derivation of the causal stage coefficient. SCDB-LSTM separately encodes the HI-derived state and multidomain vibration features, integrates their temporal evolution and uses the causal stage coefficient to condition the RUL regression head.

The coupling is sequential and explicit rather than end-to-end joint optimization: changing the HI can change the confirmed FPT, the offline target and the inference-time stage coefficient, while the predictor receives both the HI state and the stage context. Thus, the contribution lies in the specified information flow and its evaluation, not in claiming that gating, transition detection, or recurrent prediction is individually unprecedented.

The main contributions of this study are summarized as follows:

- A mechanism-informed gated health indicator is developed to organize multidomain vibration descriptors into functionally distinct degradation evidence. Fault mechanism support and cross-channel consistency are incorporated to adaptively regulate impulse-sensitive contributions, thereby separating degradation information from evidence reliability and yielding a mechanism-supported health trajectory for transition analysis.
- A causal evidence-driven transition identification method is proposed by jointly evaluating health-level deviation, trailing trend and temporal persistence. The confirmed FPT guides degradation-aware normalized RUL label construction and the causal stage coefficient, linking transition identification with subsequent RUL learning.
- A stage-conditioned dual-branch LSTM is constructed to separately encode the HI-derived state and multidomain vibration features before temporal integration. The causal stage coefficient conditions the RUL regression head, while a bearing- and stage-balanced weighting mitigates the influence of life-cycle imbalance during training.

The remainder of this paper is organized as follows. Section 2 presents the proposed framework and its three constituent modules. Section 3 reports the experimental evaluation and discussion, and Section 4 concludes the paper.

## 2. Proposed Method

**Figure 1:**
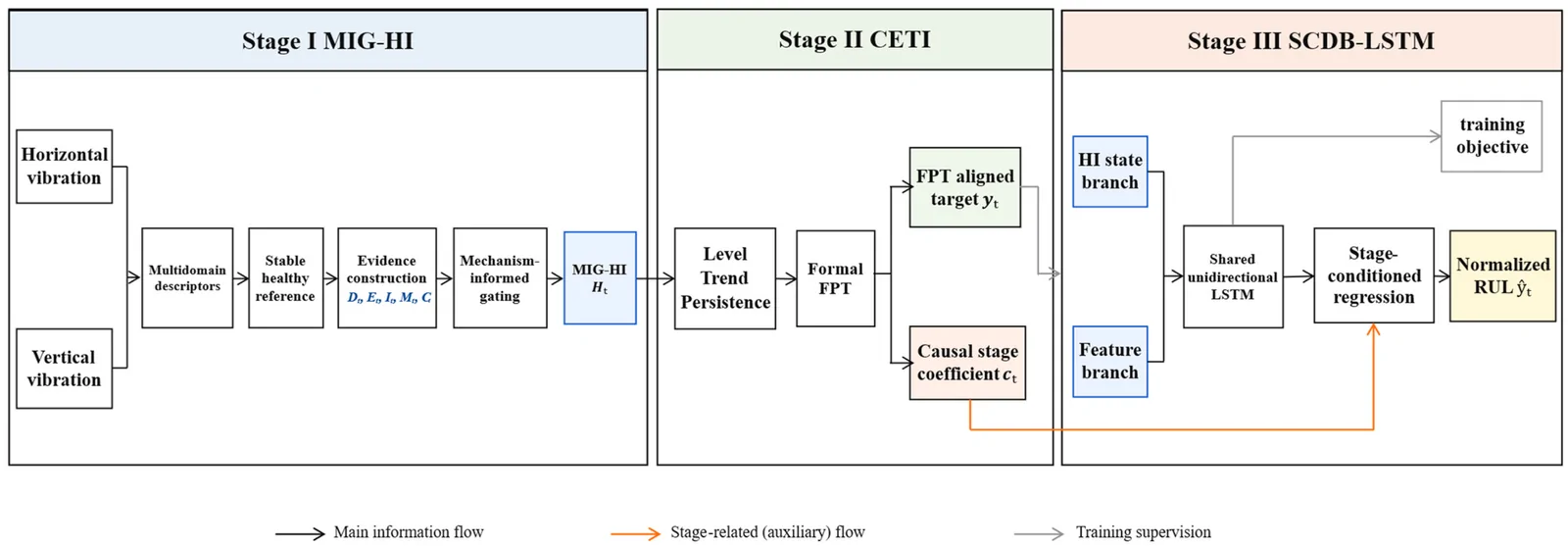
The overall framework of the proposed bearing RUL prediction method.

The proposed framework integrates health representation, causal degradation-transition identification and stage-aware temporal modeling into a sequential architecture for degradation-consistent RUL prediction. As illustrated in Figure 1, the proposed framework comprises three interconnected components: MIG-HI, CETI and SCDB-LSTM. MIG-HI establishes a robust representation of bearing health evolution from multidomain vibration information. On this basis, CETI identifies the causal transition from healthy operation to persistent degradation and derives the corresponding prediction-time reference and degradation-stage information. These outputs are subsequently incorporated into SCDB-LSTM, where the temporal evolution of the health state and complementary multidomain features is jointly modeled under stage conditioning to obtain the final RUL estimate.

### 2.1. Mechanism-Informed Gated Health Indicator

**Figure 2:**
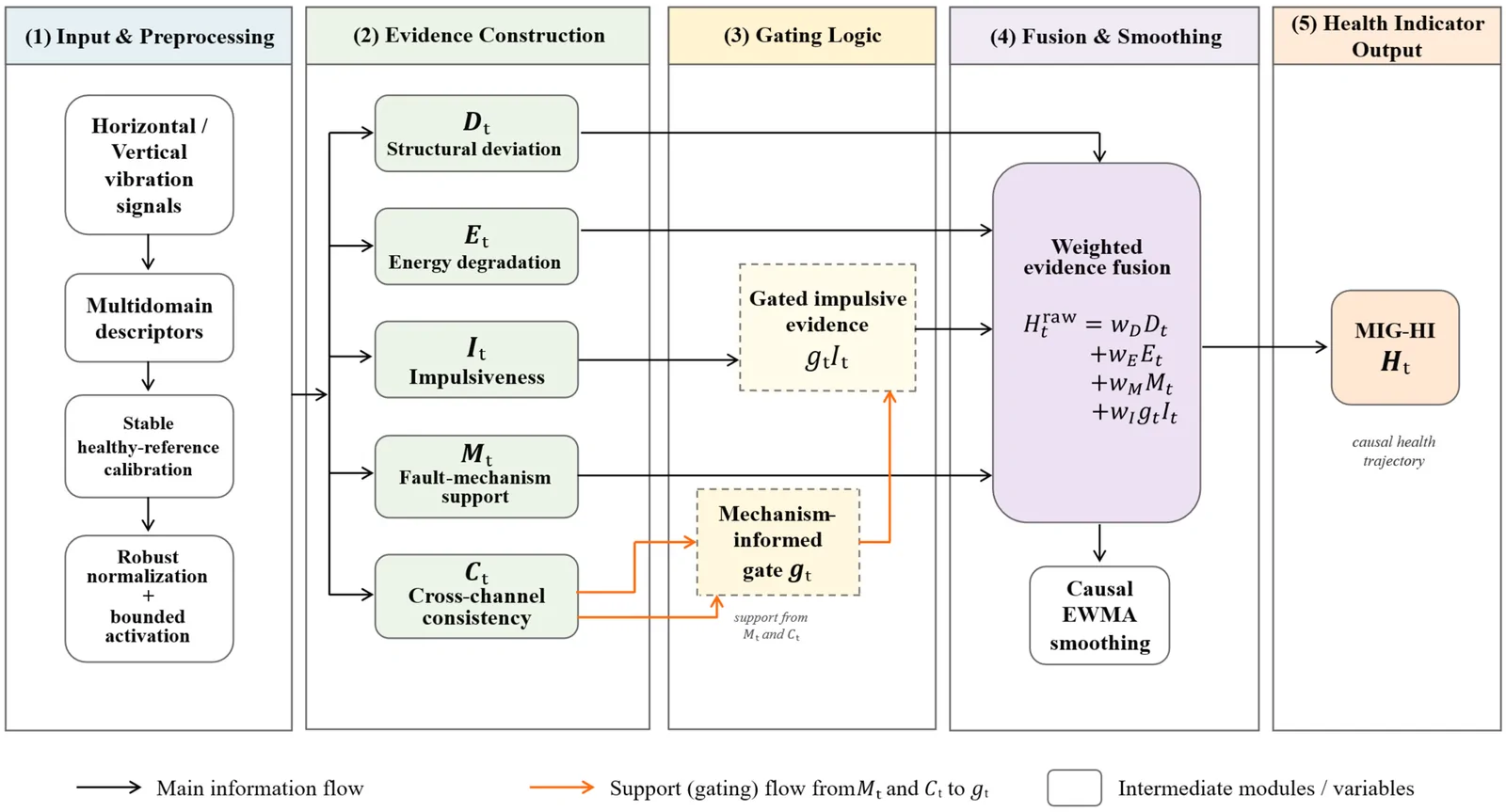
The construction of the proposed mechanism-informed gated health indicator (MIG-HI).

The MIG-HI module derives a compact, degradation-sensitive health representation from heterogeneous vibration information while reducing the influence of transient responses that lack sufficient degradation-related support. As illustrated in Figure 2, multidomain descriptors are extracted from the horizontal and vertical vibration channels and progressively transformed through healthy-reference calibration, evidence construction, mechanism-informed gating and causal smoothing. Rather than directly combining heterogeneous descriptors into a single index, the method first evaluates their departures from a stable healthy state and organizes them into physically interpretable degradation evidence. The contribution of impulse-sensitive information is then adaptively regulated according to fault mechanism and cross-channel support, yielding a health trajectory that preserves progressive degradation while attenuating insufficiently corroborated fluctuations.

Because the absolute magnitudes and fluctuation ranges of vibration descriptors vary across feature types and individual bearings, a bearing-specific healthy reference is established before constructing the degradation evidence. Specifically, an initial calibration prefix containing $N_{c}$ vibration records is reserved, within which a stable reference window of length $N_{r}$ is identified. To avoid selecting a locally fluctuating interval as the reference, a predefined set of descriptors for stability assessment is first normalized over the complete calibration prefix, and the temporal slope of each descriptor is calculated within every candidate window. For a candidate window beginning at position *s*, its stability is quantified by

(1) $$J_{s}=Q_{q_{s}}\left(\left\{\left|\beta_{s,j}\right|:j\in S_{ref}\right\}\right),$$

where $\beta_{s,j}$ denotes the least-squares slope of the j-th reference descriptor, $S_{ref}$ is the descriptor set used for stability assessment, and $Q_{q_{s}}\left(\cdot \right)$ denotes the prescribed quantile operator. The stable healthy reference is then selected as

(2) $$s^{*}=\underset{s}{argmin}J_{s},B_{ref}=\left\{s^{*},\ldots ,s^{*}+N_{r}-1\right\}.$$

This criterion favors an interval in which most reference descriptors exhibit weak temporal variation, thereby providing a more stable basis for subsequent degradation assessment than an arbitrarily predefined initial segment.

Once the stable reference window is determined, each descriptor involved in evidence construction is standardized relative to its bearing-specific healthy distribution. To reduce sensitivity to occasional outliers, the reference center and scale are estimated using the median and median absolute deviation (MAD),

(3) $$\mu_{j}=\underset{t\in B_{ref}}{median}x_{t,j},$$

(4) $$\sigma_{j}=1.4826\underset{t\in B_{ref}}{median}\left|x_{t,j}-\mu_{j}\right|,$$

The normalized deviation is subsequently obtained as

(5) $$z_{t,j}=\mathrm{clip}\left(\frac{x_{t,j}-\mu_{j}}{\sigma_{j}+\varepsilon },-z_{max},z_{max}\right).$$

For positive-valued descriptors with large dynamic ranges, including energy-, impulsiveness- and fault-frequency-related quantities, a logarithmic transformation is applied before normalization. The standardized deviations are then mapped into bounded activation responses. Descriptors for which deviations in either direction may indicate a state change are transformed using

(6) $$a_{t,j}^{\pm }=\mathrm{clip}\left(\frac{\left|z_{t,j}\right|-\theta_{a}}{w_{a}},0,1\right),$$

whereas descriptors expected to increase with degradation are transformed as

(7) $$a_{t,j}^{+}=\mathrm{clip}\left(\frac{z_{t,j}-\theta_{a}}{w_{a}},0,1\right).$$

Here, $\theta_{a}$ determines the deviation required for activation and $w_{a}$ controls the transition toward saturation. These transformations place heterogeneous descriptors on a comparable bounded scale while retaining their different degradation directions.

As summarized in Table 1, the activated responses are organized into five complementary evidence groups: structural deviation $D_{t}$, energy-related degradation $E_{t}$, impulsiveness $I_{t}$, fault mechanism support $M_{t}$ and cross-channel consistency $C_{t}$. Structural evidence represents the overall departure of statistical and distributional characteristics from the healthy state and is obtained by averaging the corresponding responses,

(8) $$D_{t}=\frac{1}{\left|S_{D}\right|}\sum_{j\in S_{D}}a_{t,j}^{\pm }.$$

Energy-, impulsiveness- and fault-mechanism-related responses may instead be concentrated in a subset of degradation-sensitive descriptors. An upper-fraction aggregation operator is therefore introduced as

(9) $$U_{\rho }\left(v_{t}\right)=\frac{1}{K}\sum_{k=1}^{K}v_{t,\left(k\right)},K=\left\lceil \rho \cdot dim\left(v_{t}\right)\right\rceil .$$

where $vt,\left(1\right)\geq \cdot \cdot \cdot \geq vt,\left(p\right)$ denote the responses sorted in descending order. Accordingly,

(10) $$E_{t}=U_{\rho_{E}}\left(a_{t}^{E}\right),I_{t}=U_{\rho_{I}}\left(a_{t}^{I}\right),M_{t}=U_{\rho_{M}}\left(a_{t}^{M}\right).$$

This aggregation emphasizes pronounced degradation-sensitive responses without allowing a large number of weakly activated descriptors to dilute their contribution. In particular, $E_{t}$ reflects changes in vibration-, envelope- and frequency-domain energy, $I_{t}$ characterizes localized impulsive behavior, and $M_{t}$ quantifies the relative concentration of envelope-spectrum energy around BPFO, BPFI, BSF and FTF and their harmonics.

Cross-channel consistency is treated differently from the preceding degradation evidence because its primary role is corroborative rather than quantitative. The relative agreement between the horizontal and vertical channels is characterized using waveform correlation together with the symmetry of RMS, peak-to-peak amplitude, spectral energy and envelope energy. For a positive horizontal-to-vertical ratio $r_{t}^{\left(l\right)}$, the corresponding symmetry score is defined as

(11) $$c_{t}^{\left(l\right)}=\exp\left(-\left|lnr_{t}^{\left(l\right)}\right|\right).$$

The score approaches unity when the relative responses of the two channels are comparable and decreases as their discrepancy increases. Together with the absolute inter-channel correlation, these measures form the consistency vector $c_{t}$, from which

(12) $$\begin{array}{c} C_{t}=U_{\rho_{C}}\left(c_{t}\right). \end{array}$$

is obtained. Therefore, $C_{t}$ does not directly quantify degradation severity; instead, it indicates whether an observed abnormal response is supported across sensing directions.

The mechanism-informed gate constitutes the key distinction between MIG-HI and conventional fixed evidence fusion. Although impulsive descriptors are highly responsive to localized bearing defects, pronounced impulsive excursions may also be induced by isolated shocks, transient disturbances, or channel-specific abnormalities. A large *I_t_* alone is therefore insufficient to establish that the observed response is associated with persistent bearing degradation. As depicted in Figure 2, the proposed gate evaluates the impulsive evidence jointly with fault mechanism support *M_t_* and cross-channel consistency $C_{t}$, and defines

(13) $$g_{t}=g_{min}+\left(1-g_{min}\right)\sigma \left(\frac{\lambda_{M}M_{t}+\lambda_{C}C_{t}-\theta_{g}}{T_{g}}\right).$$

where $\sigma \left(\cdot \right)$ denotes the sigmoid function, $g_{min}$ specifies the minimum retained contribution, $\lambda_{M}$ and $\lambda_{C}$ determine the relative contributions of the two supporting evidences, $\theta_{g}$ is the gating threshold, and $T_{g}$ controls the transition sharpness. Stronger mechanism-related and cross-channel support drives $g_{t}$ toward unity, allowing the impulsive evidence to contribute more fully. Conversely, when such support is weak, the contribution of $I_{t}$ is attenuated while a nonzero minimum gate prevents potentially informative impulsive changes from being completely discarded.

This gate encodes corroboration rather than a physical guarantee. A localized defect may produce unequal responses in the two sensing directions, while a common disturbance may affect both channels. Consequently, cross-channel agreement alone does not establish a fault, and gate-based attenuation must be distinguished from experimentally demonstrated robustness to specified disturbances.

The raw health indicator is subsequently constructed as

(14) $$\begin{array}{c} H_{t}^{raw}=w_{D}D_{t}+w_{E}E_{t}+w_{M}M_{t}+w_{I}g_{t}I_{t}, \end{array}$$

subject to

(15) $$\begin{array}{c} w_{D}+w_{E}+w_{M}+w_{I}=1,w_{D},w_{E},w_{M},w_{I}\geq 0. \end{array}$$

Notably, *C_t_* does not directly enter the weighted health-index fusion. Instead, it acts together with $M_{t}$ as supporting information for regulating the contribution of $I_{t}$, thereby separating direct degradation evidence from evidence reliability. Finally, the record-wise fluctuations of the raw indicator are reduced using a recursive exponentially weighted moving average,

(16) $$H_{t}=\alpha_{H}H_{t}^{raw}+\left(1-\alpha_{H}\right)H_{t-1},$$

where $\alpha_{H}$ ∈ (0, 1] denotes the smoothing coefficient. Once the initial calibration interval has been completed, the healthy-reference statistics remain fixed, and the subsequent update of $H_{t}$ requires only the current vibration observation and the preceding HI value. By differentiating the functional roles of the evidence components and adaptively regulating impulse-sensitive contributions according to fault mechanism and cross-channel support, MIG-HI implements a role-specific fusion strategy instead of uniformly combining heterogeneous degradation evidence. The resulting MIG-HI therefore provides the causal health trajectory used by the subsequent degradation-transition identification and RUL prediction modules.

### 2.2. Causal Evidence-Driven Transition Identification

**Figure 3:**
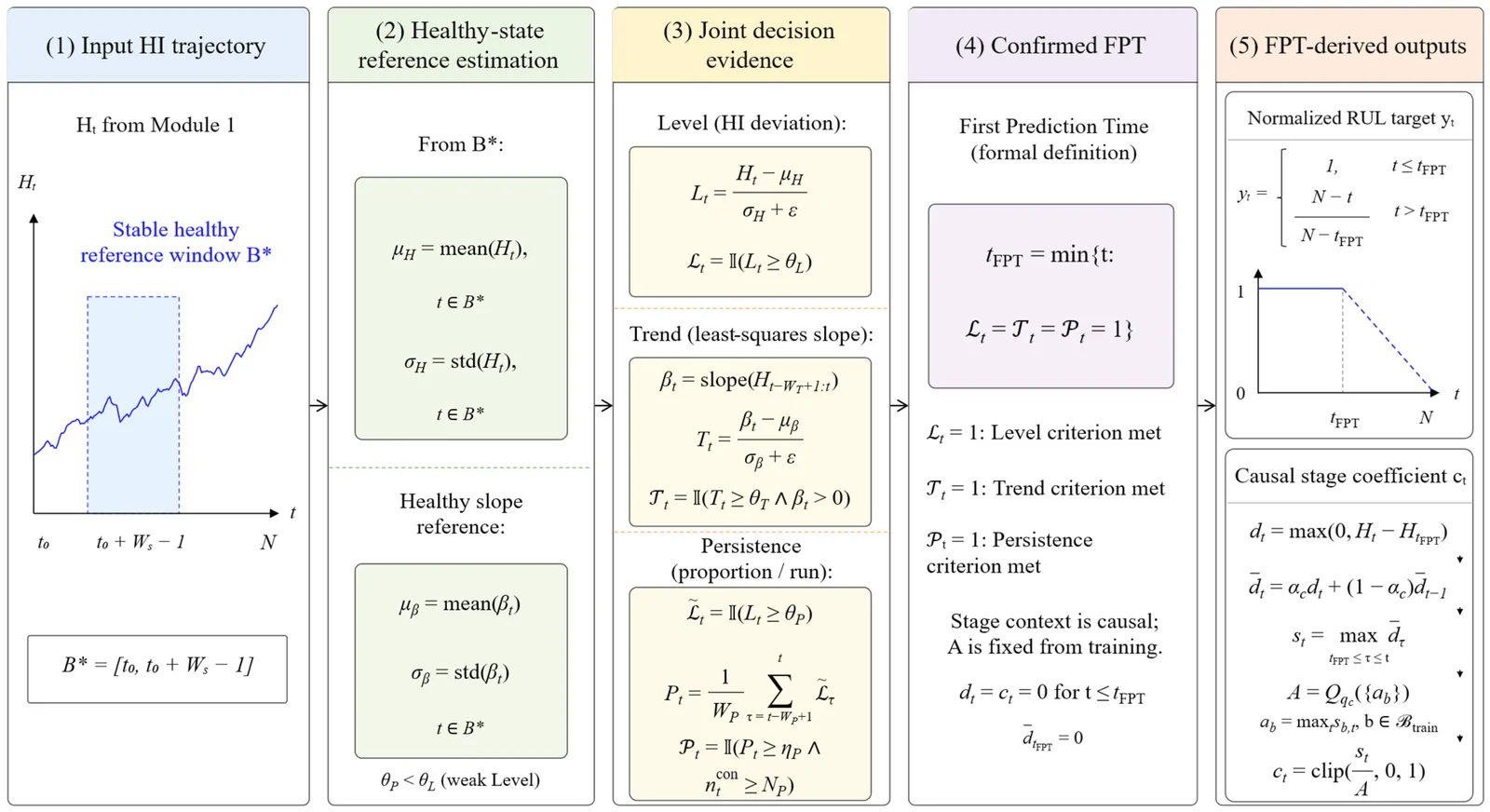
CETI information flow for formal FPT identification and FPT-derived outputs.

The CETI module identifies the transition of a bearing from healthy operation to sustained degradation from the MIG-HI trajectory without relying on future observations. A large instantaneous increase in the health indicator is insufficient to confirm this transition because transient disturbances may produce similar responses without indicating that sustained degradation has developed. As illustrated in Figure 3, CETI evaluates health-state evolution from three complementary perspectives: the degree of departure from the healthy distribution, the direction and strength of recent temporal evolution, and the temporal persistence of abnormal behavior. These aspects are represented by Level, Trend and Persistence evidence, respectively. Their joint decision determines the earliest time at which sustained degradation can be causally confirmed, which is defined as the formal first prediction time (FPT).

Level evidence quantifies the instantaneous departure of the current health state from the healthy reference. Let $\mu_{H}$ and $\sigma_{H}$ denote the center and scale of the MIG-HI within the healthy-reference interval, respectively. The standardized magnitude-deviation score is defined as

(17) $$L_{t}=\frac{H_{t}-\mu_{H}}{\sigma_{H}+\varepsilon },$$

where $H_{t}$ denotes the MIG-HI at record t, and *ε* is a small constant introduced for numerical stability. A strong Level condition is further defined as

(18) $$L_{t}=I\left(L_{t}\geq \theta_{L}\right),$$

where $\theta_{L}$ is the minimum standardized deviation required for the current health state to be regarded as significantly different from the healthy state. Evaluating the HI relative to each bearing’s own healthy distribution avoids imposing a common absolute threshold on bearings with different baseline magnitudes and fluctuation ranges. However, $L_{t}=1$ indicates only a significant instantaneous magnitude anomaly and cannot by itself establish that sustained degradation has developed.

To determine whether the current departure is accompanied by temporal evolution toward degradation, the recent HI change is evaluated over a trailing window of length $W_{T}$. The local trend at record *t* is represented by the least-squares slope

(19) $$\beta_{t}=slope\left(H_{t-W_{T}+1:t}\right).$$

The raw slope is then standardized relative to the slope distribution obtained from the healthy-reference interval. Let $\mu_{\beta }$ and $\sigma_{\beta }$ denote the center and scale of the corresponding reference slopes, respectively. The standardized Trend score is defined as

(20) $$T_{t}=\frac{\beta_{t}-\mu_{\beta }}{\sigma_{\beta }+\varepsilon }.$$

A positive degradation-trend condition is further defined as

(21) $$T_{t}=I\left(T_{t}\geq \theta_{T}\wedge \beta_{t}>0\right),$$

where $\theta_{T}$ controls the significance required relative to normal slope fluctuations in the healthy state. The explicit $\beta_{t}>0$ constraint ensures that only an HI trend increasing in the degradation direction is accepted as valid trend evidence. Trend evidence therefore distinguishes an emerging degradation trajectory from a large but isolated health-state anomaly.

Persistence evidence is introduced to determine whether abnormal behavior has become temporally sustained. Instead of directly reusing the strong Level condition, a weaker magnitude-deviation condition is first defined as

(22) $${\overset{\sim }{L}}_{t}=I\left(L_{t}\geq \theta_{P}\right),\theta_{P}<\theta_{L}.$$

The weaker condition captures recurrent moderate health deviations before or near the formal degradation transition. Within a trailing window of length $W_{P}$, the proportion of weakly abnormal records is defined as

(23) $$P_{t}=\frac{1}{W_{P}}\sum_{\tau =t-W_{P}+1}^{t}{\overset{\sim }{L}}_{\tau }.$$

The Persistence condition is then defined as

(24) $$P_{t}=I\left(P_{t}\geq \eta_{P}\wedge n_{t}^{con}\geq N_{P}\right),$$

where $\eta_{P}$ is the required proportion of weak abnormalities, $n_{t}^{con}$ is the maximum consecutive length of weakly abnormal states within the current persistence window and $N_{P}$ is the minimum number of consecutive abnormal records. Jointly imposing the proportion and continuity constraints prevents isolated anomalies from being misinterpreted as sustained degradation without requiring every sample in the window to reach the strong Level threshold. The functions of the three causal evidence types are summarized in Table 2.

The degradation transition is formally confirmed when all three causal conditions are satisfied simultaneously. The FPT is therefore defined as

(25) $$t_{FPT}=min\left\{t:L_{t}=T_{t}=P_{t}=1\right\}.$$

All three criteria depend only on the current and previously available observations; consequently, the decision made at record t does not require subsequent HI samples. The resulting $t_{FPT}$ is the earliest time at which sustained degradation is causally confirmed, rather than the exact physical onset of bearing damage. If an earlier transition location is backtracked for visualization or retrospective analysis, that location is not used to construct the subsequent supervision target or stage information, both of which are referenced to the formal $t_{FPT}$.

During supervised model training, the formal FPT is used to construct the degradation-aware normalized RUL label $y_{t}$. The nominal operating stage is assigned the maximum normalized RUL value, while the post-FPT interval describes the progressive consumption of the remaining useful life as degradation evolves toward failure. Accordingly, $y_{t}$ is defined as:

(26) $$y_{t}=\left\{\begin{array}{cc} 1, & t\leq t_{FPT},\\ \frac{N-t}{N-t_{FPT}}, & t>t_{FPT}, \end{array}\right.$$

where *N* is the total number of records in the complete run-to-failure sequence. Thus, $y_{t}$ remains equal to 1 before the formal FPT and decreases linearly from 1 to 0 over the subsequent degradation interval. This construction provides a degradation-aware supervision trajectory for RUL learning, with the formal FPT serving as the transition reference between the nominal and degradation stages. The complete sequence length is used only for offline label construction and performance evaluation and is not provided to the prediction model during inference.

The supervised quantity is a degradation-aligned normalized prognostic target, not the physical remaining operating time over the entire life cycle. Physical remaining time decreases before the FPT, whereas this target remains constant by design. Its plateau reduces the emphasis on differences within nominal operation. A dimensionless prediction cannot be converted into minutes by multiplying it by an unknown test-bearing post-FPT lifetime; that quantity is unavailable during deployment. Throughout the experimental results, normalized RUL refers to this target and reported errors are dimensionless.

In addition to defining the supervision boundary, the formal FPT is used to construct a continuous stage coefficient describing post-FPT degradation progress. Taking the HI at the FPT as a local reference, a non-negative health-deviation quantity is first defined as

(27) $$d_{t}=\left\{\begin{array}{cc} 0, & t\leq t_{FPT},\\ max\left(0,H_{t}-H_{t_{FPT}}\right), & t>t_{FPT}. \end{array}\right.$$

Recursive smoothing is then applied to reduce short-term fluctuations while retaining causal online updating:

(28) $${\bar{d}}_{t}=\alpha_{c}d_{t}+\left(1-\alpha_{c}\right){\bar{d}}_{t-1},{\bar{d}}_{t_{FPT}}=0.$$

where *α_c_* is the stage-smoothing coefficient. A running maximum is subsequently used to obtain a monotonically nondecreasing degradation-stage trajectory:

(29) $$s_{t}=\underset{t_{FPT}\leq \tau \leq t}{max}{\bar{d}}_{\tau }.$$

This operation prevents a temporary decrease in the HI from producing a physically implausible reversal in the inferred degradation stage.

To improve the comparability of stage representations across bearings, a common normalization scale is estimated using training bearings only. For each training bearing *b*, define

(30) $$a_{b}=\underset{t}{max}s_{b,t}.$$

The common stage scale is then obtained as

(31) $$A=Q_{q_{c}}\left(\left\{a_{b}:b\in B_{train}\right\}\right).$$

where $Q_{q_{c}}\left(\cdot \right)$ is the prescribed quantile operator and $B_{train}$ is the current set of training bearings. Once estimated from the training data, *A* remains fixed for validation and test bearings. The causal stage coefficient is finally defined as

(32) $$c_{t}=clip\left(\frac{s_{t}}{A},0,1\right).$$

Consequently, $c_{t}$ is zero before the formal FPT and increases as health deviation accumulates after the FPT. Importantly, $c_{t}$ is a normalized relative degradation-stage indicator rather than the percentage of physical lifetime consumed. For a test bearing, its update uses only observations available up to the current record and the stage scale fitted previously from the training bearings; no future test samples are required.

The degradation-aware normalized RUL label $y_{t}$ provides the regression supervision used for offline training, whereas the causal stage coefficient $c_{t}$ provides stage-conditioning information during prediction. The subsequent SCDB-LSTM uses these quantities together with the degradation representations for RUL prediction, thereby exploiting supervision aligned with the degradation transition without introducing future information from the test bearing into stage-conditioned inference.

### 2.3. Stage-Conditioned Dual-Branch LSTM for RUL Prediction

**Figure 4:**
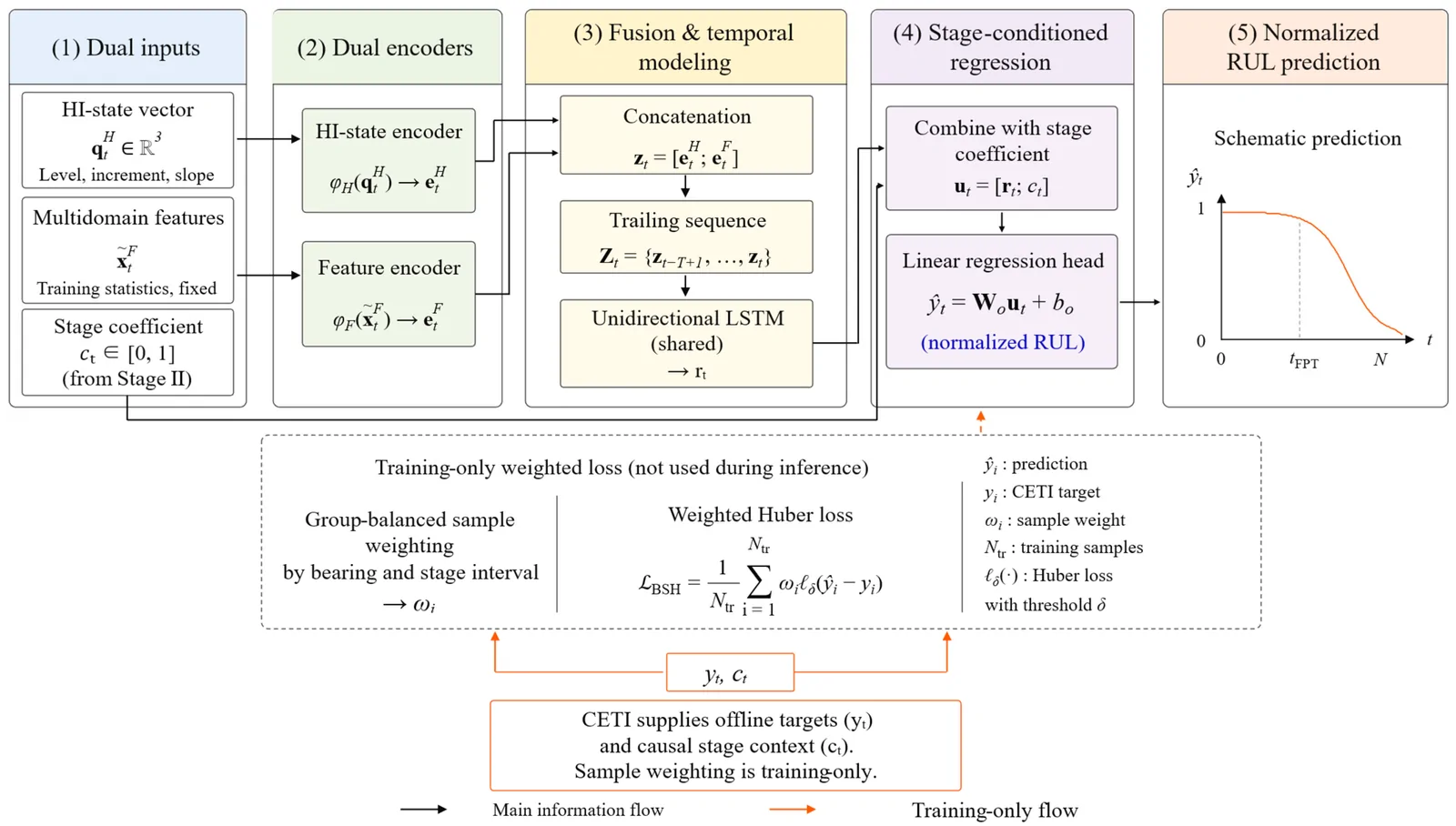
SCDB-LSTM architecture and training-only weighted loss design.

The SCDB-LSTM jointly models the compact health-state evolution provided by MIG-HI and the complementary degradation information retained in multidomain vibration features. Although the health indicator provides a stable and interpretable representation of the overall degradation process, compressing heterogeneous vibration information into a single health trajectory inevitably discards some local variations that remain valuable for RUL estimation. Conversely, directly modeling the complete multidomain feature sequence without an explicit health-state representation may increase the influence of redundant features and local fluctuations. As shown in Figure 4, SCDB-LSTM therefore employs two branch-specific mappings to encode the HI state and multidomain vibration features separately, followed by a shared unidirectional LSTM [21] that models their joint temporal evolution after representation fusion. The causal stage coefficient $c_{t}$ obtained by CETI is subsequently introduced as stage-conditioning information for degradation-aware normalized RUL estimation.

The HI-state branch characterizes the health condition using the current MIG-HI level, its first-order increment and the recent temporal trend. Specifically, the causal HI state for record *t* is defined as

(33) $$q_{t}^{H}=\left[\begin{array}{c} H_{t}\\ \Delta H_{t}\\ \beta_{t}^{H} \end{array}\right],\;\;\Delta H_{t}=H_{t}-H_{t-1},$$

where $H_{t}$ represents the accumulated health-state level, $\Delta H_{t}$ describes the most recent local change, and $\beta_{t}^{H}$ is the local slope obtained by least-squares fitting of MIG-HI within a trailing window ending at the current record *t*. Because both $\Delta H_{t}$ and $\beta_{t}^{H}$ depend only on observations available up to the current time, $q_{t}^{H}$ respects the temporal information boundary. A branch-specific mapping then produces the hidden HI-state representation:

(34) $$e_{t}^{H}=ReLU\;\left[LN\;\left(W_{H}q_{t}^{H}-b_{H}\right)\right],$$

where $W_{H}$ and $b_{H}$ are learnable parameters and $LN\;\left(\cdot \right)$ denotes layer normalization. Thus, the temporal predictor receives not merely a single HI magnitude but an explicit health-state representation that simultaneously captures the accumulated degradation level, local change and recent evolution trend.

The multidomain feature branch complements the compact health-state representation by retaining local vibration-degradation information that may not be fully reflected in MIG-HI. Let $x_{t}^{F}\in R^{d_{F}}$ denote the multidomain feature vector for record t. To prevent information from validation and test bearings from entering model preprocessing, each feature dimension is standardized using statistics obtained exclusively from the training bearings:

(35) $${\tilde{x}}_{t}^{F}=\left(x_{t}^{F}-\mu_{F}^{tr}\right)⊘\left(\sigma_{F}^{tr}+\varepsilon \right),$$

where ⊘ denotes element-wise division, and $\mu_{F}^{tr}$ and $\sigma_{F}^{tr}$ are the feature-wise mean and standard deviation, respectively, estimated only from the training bearings. These statistics remain fixed during validation and testing. The standardized features are then processed by an independent branch-specific mapping:

(36) $$e_{t}^{F}=ReLU\;\left[LN\;\left(W_{F}{\tilde{x}}_{t}^{F}-b_{F}\right)\right].$$

This branch retains complementary statistical, frequency-domain, impulsive, fault-related and cross-channel information while preventing the high-dimensional feature representation from numerically dominating the low-dimensional HI state during direct fusion.

After the branch-specific representations are obtained, the two embeddings associated with the same record are concatenated to form the joint degradation representation:

(37) $$z_{t}=\left[e_{t}^{H};e_{t}^{F}\right].$$

For prediction at record *t*, the most recent *T* joint representations form the input sequence:

(38) $$Z_{t}=\left[z_{t-T+1},\ldots ,z_{t}\right].$$

SCDB-LSTM does not assign an independent recurrent network to each input branch. Instead, unified temporal modeling is performed after representation-level fusion. The shared unidirectional LSTM updates its state as

(39) $$h_{\tau }=LSTM\;\left(z_{\tau },h_{\tau -1}\right),\;\;\tau =t-T+1,\ldots ,t,$$

and the hidden state corresponding to the last record of the input window is retained as the sequence-to-one temporal representation:

(40) $$r_{t}=h_{t}.$$

Fusing the two branches before recurrent modeling allows the LSTM to learn not only temporal dependencies within each information source but also the coupled evolution between the compact health state and complementary multidomain features. The shared temporal encoder also avoids the duplicated recurrent parameters required by two separate LSTMs. Because the recurrence is unidirectional and the input window terminates at the current record t, $r_{t}$ contains no information from subsequent vibration records.

Stage-conditioning information is introduced after temporal encoding so that the historical degradation dynamics and the current degradation stage retain distinct functions. The temporal representation is concatenated with the causal stage coefficient obtained in Section 2.2:

(41) $$u_{t}=\left[r_{t};c_{t}\right].$$

A linear regression head then produces the predicted degradation-aware normalized RUL, ${\hat{y}}_{t}$:

(42) $${\hat{y}}_{t}=W_{o}u_{t}+b_{o},$$

where $W_{o}$ and $b_{o}$ are learnable regression parameters. In this structure, $r_{t}$ summarizes the observed degradation dynamics within the input history, whereas $c_{t}$ describes the relative degradation stage at the current prediction time. The stage coefficient therefore conditions the RUL mapping rather than serving as another RUL supervision target. The final regression head uses no sigmoid activation and imposes no FPT-dependent output constraint; consequently, the prediction is learned directly from the training objective and is not artificially forced to equal 1 before the FPT.

Complete run-to-failure trajectories used for training may differ substantially in length, while long pre-FPT plateaus and post-FPT degradation intervals of different durations can also cause pronounced sample-density imbalance. To prevent bearings or degradation stages with more samples from dominating model optimization, training windows are jointly grouped according to their source bearing and degradation stage. Let $b_{i}$ and $g_{i}$ denote the bearing and stage group of training sample i, respectively. The healthy interval forms a separate stage group, whereas post-FPT samples are divided into several ordered stage groups according to degradation progress. For bearing-stage group $\left(b,g\right)$, its number of training samples is defined as

(43) $$n_{b,g}=\sum_{j=1}^{N_{tr}}I\left(b_{j}=b,\;g_{j}=g\right).$$

The initial weight of sample i is inversely proportional to the number of samples in its group:

(44) $${\tilde{\omega }}_{i}=\frac{1}{n_{b_{i},g_{i}}}.$$

The weights are subsequently normalized to have a mean of 1:

(45) $$\omega_{i}=\frac{N_{tr}{\tilde{\omega }}_{i}}{\sum_{j=1}^{N_{tr}}{\tilde{\omega }}_{j}}.$$

This construction gives each observed bearing-stage group an approximately comparable total contribution to the training objective without changing the overall scale of the optimization target. The weights affect only the gradient contributions during model training and are not required for RUL inference.

To further reduce the excessive influence of a small number of large prediction residuals, the Huber loss is applied together with the balancing weights [22]. Define

(46) $$e_{i}={\hat{y}}_{i}-y_{i},$$

the per-sample loss is then

(47) $$l_{\delta }\left(e_{i}\right)=\left\{\begin{array}{ll} \frac{1}{2}e_{i}^{2}, & \left|e_{i}\right|\leq \delta ,\\ \delta \left(\left|e_{i}\right|-\frac{1}{2}\delta \right), & \left|e_{i}\right|>\delta , \end{array}\right.$$

where δ controls the transition between quadratic and linear penalties. The final bearing-stage weighted Huber training objective is defined as

(48) $$L_{BSH}=\frac{1}{N_{tr}}\sum_{i=1}^{N_{tr}}\omega_{i}l_{\delta }\;\left({\hat{y}}_{i}-y_{i}\right)$$

The Huber term reduces the influence of a small number of abnormally large residuals, whereas $\omega_{i}$ compensates for the uneven representation of different bearings and degradation stages in the training data. Stage grouping and sample weights are determined only from the training data in the current experimental fold and affect only gradient-based model training.

In summary, SCDB-LSTM integrates compact HI evolution, complementary multidomain degradation information and causal degradation-stage context within a unified temporal prediction framework to establish a stage-conditioned RUL mapping. The two branch-specific mappings preserve the distinct functions of the degradation representations, the shared unidirectional LSTM models their coupled temporal evolution, and the current stage coefficient further conditions the final RUL mapping. Together with the degradation-aware normalized RUL supervision defined in Section 2.2 and the bearing-stage weighted Huber objective, the predictor maintains a clear functional separation among degradation-state representation, causal stage inference and supervised RUL learning.

## 3. Experimental Results and Discussion

The experimental evaluation is organized in three parts. First, the MIG-HI trajectories are compared with representative health indicators, including RMS-, Kurtosis- and PCA-based health indicators. Second, the degradation-transition identification results obtained by CETI are examined. Third, bearing-level RUL prediction comparisons and ablation experiments are conducted to evaluate the SCDB-LSTM predictor and the principal components of the proposed framework. All experiments follow the data-processing and evaluation protocol described below.

### 3.1. Dataset and Experimental Setup

The experiments were conducted using the rolling-bearing degradation data provided by the IEEE PHM 2012 Data Challenge and acquired on the PRONOSTIA accelerated degradation platform [23]. The complete dataset contains 17 bearing experiments performed under three constant operating conditions characterized by different rotational speeds and radial loads. In the present evaluation, a predefined subset of 13 bearing sequences was used, comprising six learning bearings and seven test bearings. The subset was specified according to the bearing identifiers before model evaluation and was not selected according to the resulting prediction performance.

For each operating condition, the first two bearings were designated as learning bearings. Bearing1_3–Bearing1_5 and Bearing2_3–Bearing2_5 were used as the test bearings under Conditions 1 and 2, respectively, while Bearing3_3 was used for testing under Condition 3. The operating conditions and bearing assignments used in this study are summarized in Table 3.

Horizontal and vertical vibration signals were synchronously recorded at 25.6 kHz. Each acquisition contains 2560 samples per channel, corresponding to 0.1 s of vibration data, and consecutive acquisitions are separated by 10 s. Each acquisition was treated as one observation record. Both channels were used for multidomain feature extraction and MIG-HI construction, including cross-channel consistency evidence.

For each observation record, multidomain descriptors were extracted from the horizontal and vertical vibration signals. These descriptors include time-domain statistics, frequency-domain shape and energy measures, envelope-domain features, characteristic-frequency energy ratios and cross-channel consistency measures. The complete descriptor set was provided to the multidomain feature branch, whereas selected descriptor subsets were used to construct MIG-HI. Because the three operating conditions have different rotational speeds, the characteristic-frequency bands were recalculated according to the rotational speed of each condition. All other feature definitions and signal-processing procedures were kept consistent across the experiments.

The three operating conditions were evaluated independently. For each condition, the two corresponding learning bearings were used for model development, and the remaining bearings were reserved exclusively for testing. Data from different operating conditions were not combined during model training. Model configurations were determined using only the learning bearings and fixed before evaluation on the corresponding test bearings. After configuration selection, the final prediction model for each condition was trained using both learning bearings. The same model architecture, implementation settings, training procedure and five random seeds were applied under all three operating conditions.

Feature-standardization parameters were estimated exclusively from the two learning bearings associated with each condition and were subsequently fixed when processing the corresponding test bearings. The bearing-specific healthy reference required by MIG-HI was established causally from the first $N_{c}$ observations of each evaluated bearing. This initial calibration used neither future observations nor RUL labels. All other learned quantities, preprocessing statistics and model-selection decisions were determined exclusively from the learning bearings. Complete-lifetime information from the test bearings was used only to construct offline reference targets and calculate evaluation metrics and was not supplied to the proposed framework during inference.

The bearing-specific reference is available only after the complete calibration prefix has been observed. Online causality therefore applies after this initialization interval. Reconstructed HI values within the prefix are retrospective calibration outputs and must not be interpreted as predictions available at their original timestamps. Healthy-reference statistics from the test prefix are a predefined unsupervised calibration step; feature standardization, the common stage scale and learned network parameters are fitted using learning bearings only. Any full-trajectory resampling used for HI evaluation is an offline diagnostic operation and is not an inference input.

Condition 1 was retained for the detailed health-representation and transition analyses to provide a focused examination of the intermediate outputs of MIG-HI and CETI. The main RUL prediction comparison was then extended to the seven test bearings from all three operating conditions. When a test bearing was processed, all preprocessing statistics and model parameters determined from the corresponding learning bearings remained fixed.

The main RUL prediction comparison was conducted on the seven test bearings and included two classical temporal models, namely LSTM and TCN, and two recent bearing-prognostic models, namely MDSCT [24] and DTA-MLP [25], together with the proposed SCDB-LSTM. All methods were evaluated using the same condition-specific learning–test division, normalized RUL target, evaluation metrics and five random seeds. The detailed configurations and implementation of the comparison methods are described in Section 3.3. The ablation experiments were restricted to Condition 1 to evaluate the contributions of the principal framework components without unnecessarily repeating identical component-level comparisons under every operating condition.

All experiments were implemented in MATLAB R2024b on a workstation equipped with an Intel Core i7-13650H CPU and an NVIDIA GeForce RTX 4060 GPU. The main implementation settings were fixed before test evaluation and are summarized in Table 4.

The evaluation framework covers health-representation quality, degradation-transition identification and RUL prediction accuracy. The health indicators are quantitatively assessed using monotonicity, trendability and prognosability [26]. For the j-th bearing, let $H_{j}\left(k\right)$ denote the HI at record k and let $N_{j}$ denote the total number of records. Monotonicity is defined as

(49) $$Mon=\frac{1}{M}\sum_{j=1}^{M}\left|\frac{1}{N_{j}-1}\sum_{k=1}^{N_{j}-1}sgn\;\left[H_{j}(k+1)-H_{j}(k)\right]\right|,$$

where M denotes the number of evaluated bearings. Since the bearings exhibit different lifetime lengths, each HI trajectory is first resampled on a common normalized lifetime axis before evaluating trendability,

(50) $$Tre=\underset{j\neq l}{min}\left|corr\;\left({\tilde{H}}_{j},{\tilde{H}}_{l}\right)\right|,$$

where ${\tilde{H}}_{j}$ denotes the resampled HI trajectory of the j-th bearing. Prognosability is defined as

(51) $$Prog=exp\;\left[-\frac{{std}_{j}\;\left(H_{j}(N_{j})\right)}{{mean}_{j}\;\left|H_{j}(1)-H_{j}(N_{j})\right|}\right].$$

Higher values of these three metrics indicate more desirable prognostic characteristics. For RUL prediction, mean absolute error (MAE) and root mean square error (RMSE) are adopted as the primary accuracy metrics,

(52) $$MAE=\frac{1}{N_{eval}}\sum_{i=1}^{N_{eval}}\left|{\hat{y}}_{i}-y_{i}\right|,$$

(53) $$RMSE=\sqrt{\frac{1}{N_{eval}}\sum_{i=1}^{N_{eval}}{\left({\hat{y}}_{i}-y_{i}\right)}^{2}}.$$

For RUL prediction, MAE and RMSE are calculated on the degradation-aware normalized RUL scale defined in Section 2.2 and are therefore dimensionless. The same target definition and evaluation rules are applied to all comparison methods. MAE and RMSE are first calculated separately for each test bearing and then arithmetically averaged across the test bearings within each operating condition. The overall results are obtained by averaging the bearing-level metrics across all seven test bearings so that bearings with longer sequences do not dominate the evaluation. Each model is independently trained using five random seeds, and the resulting metrics are reported as the mean ± standard deviation.

### 3.2. Evaluation of Health Representation and Transition Identification

Before evaluating the final RUL predictor, the intermediate outputs of MIG-HI and CETI were examined under Condition 1. This analysis addresses two questions: whether MIG-HI provides a degradation-sensitive health representation and whether CETI can identify a causal transition from nominal operation to persistent degradation. All five bearings under Condition 1 were included in the quantitative analysis. Parameters determined from Bearing1_1 and Bearing1_2 were kept fixed when the remaining bearings were processed.

MIG-HI was compared with RMS-HI, Kurtosis-HI and PCA-HI. RMS-HI represents the overall vibration-energy level, Kurtosis-HI emphasizes impulsive behavior and PCA-HI provides a data-driven combination of multidomain descriptors. These indicators therefore represent complementary reference strategies for assessing the proposed mechanism-informed construction. All four indicators were evaluated using the same observation sequences and the metric definitions given in Section 3.1.

**Figure 5:**
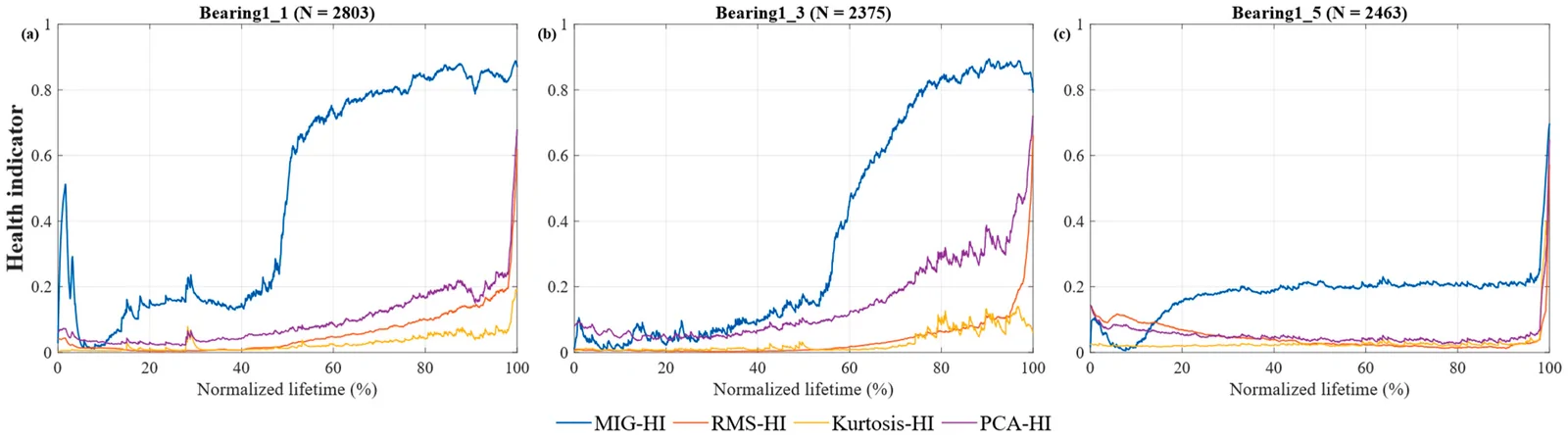
Complete-life trajectories of RMS-HI, Kurtosis-HI, PCA-HI and MIG-HI for three bearings with different degradation patterns under Condition 1: (**a**) Bearing1_1; (**b**) Bearing1_3; and (**c**) Bearing1_5.

To examine whether the observed health-representation characteristics were specific to a single degradation trajectory, three bearings with distinct degradation patterns were selected for visualization. Bearing1_1 exhibits an intermediate-life transition accompanied by several early transient excursions, Bearing1_3 presents a relatively extended progressive-degradation interval and Bearing1_5 is characterized by an abrupt late-life change. Figure 5 compares the complete-life trajectories of the four health indicators for these three bearings.

Figure 5 demonstrates that the health-indicator trajectories vary considerably across different bearing degradation patterns. For Bearing1_1, MIG-HI exhibits several early transient excursions that subsequently return toward the healthy-state level. A sustained increase emerges near the middle of the lifetime and develops into a relatively stable high-level degradation region. Bearing1_3 presents a more continuous progressive evolution, with MIG-HI increasing gradually before reaching a late-life plateau. By contrast, Bearing1_5 exhibits limited progressive evidence over most of its lifetime, followed by an abrupt increase close to the failure endpoint. This late response is also observed in the comparison indicators, suggesting that the available vibration observations contain only weak early degradation evidence for this bearing.

The comparison indicators exhibit different sensitivities to these degradation trajectories. RMS-HI remains relatively low over most of the lifetime and responds mainly to pronounced late-stage energy growth. Kurtosis-HI is more sensitive to localized impulsive variations but shows greater fluctuation in the late-life region. PCA-HI captures gradual multidomain changes, although its response magnitude and temporal evolution remain bearing-dependent. MIG-HI provides an earlier and more sustained representation of progressive degradation for Bearing1_1 and Bearing1_3 while retaining the abrupt late-life pattern of Bearing1_5. Nevertheless, the early transient excursions observed for Bearing1_1 demonstrate that an isolated HI increase should not be interpreted directly as a persistent degradation transition.

Because visual trajectory comparison alone cannot provide a complete assessment of health-representation quality, monotonicity, trendability and prognosability were further calculated across all five bearings under Condition 1. The quantitative results are summarized in Table 5. Because the three metrics characterize different properties of a health representation, they are considered jointly rather than treating any individual metric as the sole evaluation criterion.

As reported in Table 5, MIG-HI obtains monotonicity, trendability and prognosability values of 0.1575, 0.4967 and 0.8560, respectively. Its trendability exceeds that of RMS-HI by 0.2798, corresponding to a relative increase of approximately 129.00%, indicating a more consistent degradation tendency across bearings with different lifetime characteristics. This result is consistent with the role-specific evidence organization and mechanism-informed regulation adopted in MIG-HI, which aim to preserve degradation-related information while reducing the influence of insufficiently supported impulsive responses. However, MIG-HI exhibits the lowest monotonicity among the compared indicators, reflecting the retention of local fluctuations and transient recovery rather than imposing an explicitly monotonic trajectory. Its prognosability is also lower than that of PCA-HI and RMS-HI. Therefore, the results support a specific advantage of MIG-HI in cross-bearing trajectory consistency rather than uniform superiority across all HI quality metrics. The remaining nonmonotonic behavior further motivates the use of multiple forms of persistent evidence in the subsequent transition-identification stage.

The bearing-dependent trajectories in Figure 5 further indicate that a degradation transition cannot be determined reliably from an isolated HI excursion or a fixed position in the bearing lifetime. In particular, the early transient responses of Bearing1_1 and the abrupt late-life change of Bearing1_5 require the transition decision to distinguish temporary fluctuations from persistent degradation. CETI was therefore applied to MIG-HI by jointly evaluating level, trailing-trend and persistence evidence. The formal FPT was identified as the earliest searchable observation at which the three causal conditions were simultaneously satisfied.

**Figure 6:**
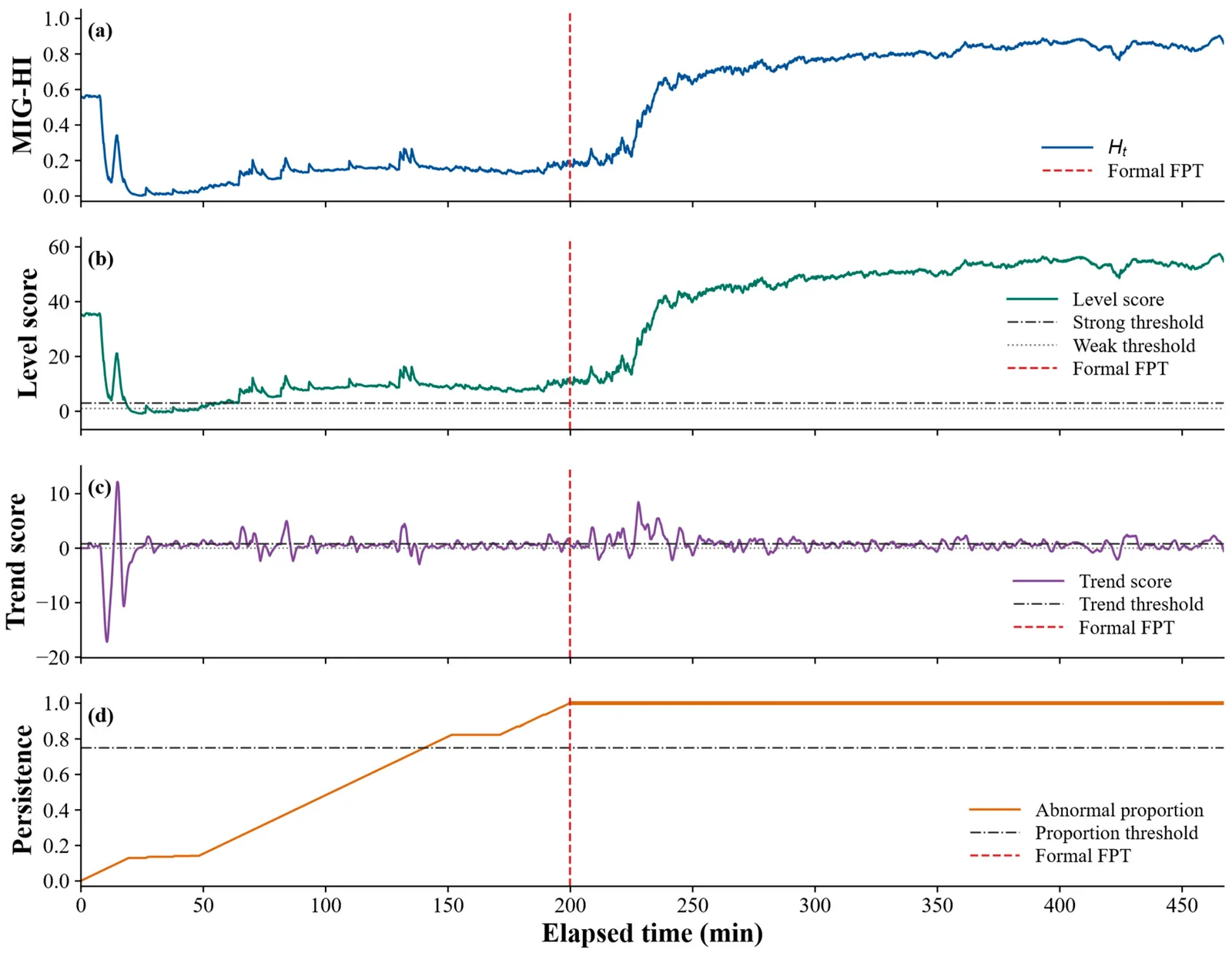
The representative CETI transition identification process based on the MIG-HI trajectory: (**a**) MIG-HI, (**b**) standardized Level evidence, (**c**) Trend evidence and (**d**) Persistence evidence. The vertical dashed line denotes the formal FPT.

Figure 6 illustrates the CETI transition identification process for Bearing1_1. The standardized level evidence characterizes the deviation of the current health state from its healthy reference, the trailing-trend evidence describes the recent direction of HI evolution, and the persistence evidence evaluates whether the abnormal behavior has been sustained over the specified causal window.

As shown in Figure 6, temporary activation of an individual evidence component does not immediately trigger the transition decision. The formal FPT is confirmed only when the current health state has departed sufficiently from its reference level, the recent HI trajectory indicates increasing degradation, and this behavior satisfies the persistence requirement. Because all three evidence quantities are calculated using only current and historical observations, the identified transition does not rely on future information.

The formal FPTs identified for the five bearings under Condition 1 are summarized in Table 6. The relative FPT position is calculated as $100t_{\mathrm{FPT}}/N$, where N denotes the total number of observations in the corresponding bearing trajectory. The number of post-FPT observations is calculated as $N-t_{\mathrm{FPT}}$, consistent with the target definition in Section 2.2.

Based on the relative FPT position and the duration of the post-FPT interval, the observed degradation trajectories are descriptively divided into two forms. Progressive degradation refers to trajectories in which persistent degradation is confirmed sufficiently before failure and continues over an observable interval. Abrupt degradation refers to trajectories that remain relatively stable over most of the bearing lifetime and exhibit persistent degradation evidence only near the failure endpoint. This distinction characterizes the temporal form of the observed degradation trajectory and does not imply a separate physical failure mechanism.

The results in Table 6 reveal two characteristic forms of bearing degradation. For the progressively degrading bearings, including Bearing1_1, Bearing1_3 and Bearing1_4, the formal FPT occurs sufficiently before the failure endpoint, leaving an observable interval in which the degradation evidence develops continuously. Their trajectories therefore provide an extended basis for stage representation and subsequent RUL regression. By contrast, Bearing1_2 and Bearing1_5 exhibit abrupt degradation. Their formal FPTs are located near the end of the corresponding lifetimes, and only a limited number of observations remain after the transition. This result is consistent with the late changes observed in their health-indicator trajectories and indicates that only limited advance warning is available for these bearings.

The coexistence of progressive and abrupt observed trajectories motivates bearing-specific transition references. However, a late formal FPT may reflect both weak early evidence and delay introduced by gating, smoothing, thresholds and persistence requirements. Without an independently annotated damage-onset time, these contributions cannot be separated. The current trajectories therefore do not establish that delayed confirmation is always preferable to an earlier warning.

The identified FPT should therefore be interpreted as the earliest causal confirmation of persistent degradation under the reported CETI configuration rather than as the exact physical onset of bearing damage. Because the PHM 2012 dataset does not provide ground-truth annotations for damage initiation, the degradation-pattern classification is used to describe the observed temporal trajectories rather than to infer the underlying physical failure modes. The effects of FPT-aligned supervision and causal stage conditioning are further examined through the prediction comparison and ablation experiments in Section 3.3 and Section 3.4.

For Bearing1_2 and Bearing1_5, the reported FPT positions are approximately 93.0% and 97.4% of the record counts. The inclusive post-FPT sample counts in Table 6 are 62 and 64; with a 10 s interval, the elapsed time from the FPT record to the final recorded sample is approximately 610 s and 630 s, respectively. These short observable intervals constrain the available warning horizon and should not be interpreted as proof that physical damage began only at the detected FPT.

### 3.3. RUL Prediction Performance and Comparison

Following the condition-specific experimental protocol described in Section 3.1, the RUL prediction performance of SCDB-LSTM was evaluated on seven test bearings from the three operating conditions and compared with four representative prognostic methods.

The comparison included two classical temporal models and two recent bearing-prognostic methods. LSTM was selected as a recurrent baseline for sequence-based RUL regression, while TCN was included to represent causal convolution-based temporal modeling. MDSCT [24] combines multiscale convolutional feature extraction with an enhanced Transformer architecture, whereas DTA-MLP [25] uses mixed multilayer perceptrons and dynamic temporal attention for bearing RUL prediction. The latter two methods represent recent convolution–Transformer and attention–MLP prognostic architectures that have been evaluated on the PHM 2012 dataset.

For the vanilla LSTM and TCN baselines, the HI-derived state and multidomain descriptors were concatenated at each observation and supplied as a common input sequence. MDSCT and DTA-MLP were reimplemented following their published principal feature-processing and temporal-modeling designs, with the input and output dimensions adapted to the present dataset and normalized RUL target. All methods used the same condition-specific learning–test division, target definition, evaluation metrics and five random seeds. Hyperparameters and model configurations were determined using only the corresponding learning bearings and were fixed before test evaluation.

For each random seed, MAE and RMSE were first calculated separately for every test bearing. Condition-level results were obtained by averaging the three test bearings under Conditions 1 and 2, whereas the Condition 3 results correspond directly to Bearing3_3. The overall results were calculated by averaging across all seven test bearings so that each bearing contributed equally regardless of its sequence length. Table 7 reports the mean and standard deviation across the five random seeds.

Table 7 presents condition-level errors for the five methods. The proposed method has the lowest tabulated mean MAE and RMSE under Conditions 1 and 3, whereas DTA-MLP has the lowest values under Condition 2. Thus, the table does not support uniform superiority across operating conditions. Overall performance must be computed from the seven bearing-level results for each seed before calculating the mean and standard deviation across seeds.

The methods differ in both their representation structure and use of stage information. LSTM and TCN use concatenated HI-state and multidomain inputs, whereas the proposed predictor preserves separate input mappings and introduces a causal stage coefficient at the regression head. MDSCT and DTA-MLP represent recent convolution–Transformer and attention–MLP designs. Accordingly, the comparison evaluates the complete implemented pipelines; it cannot isolate architectural superiority from the effect of additional stage information. Input-matched controls and complete implementation settings are required to separate these effects.

**Figure 7:**
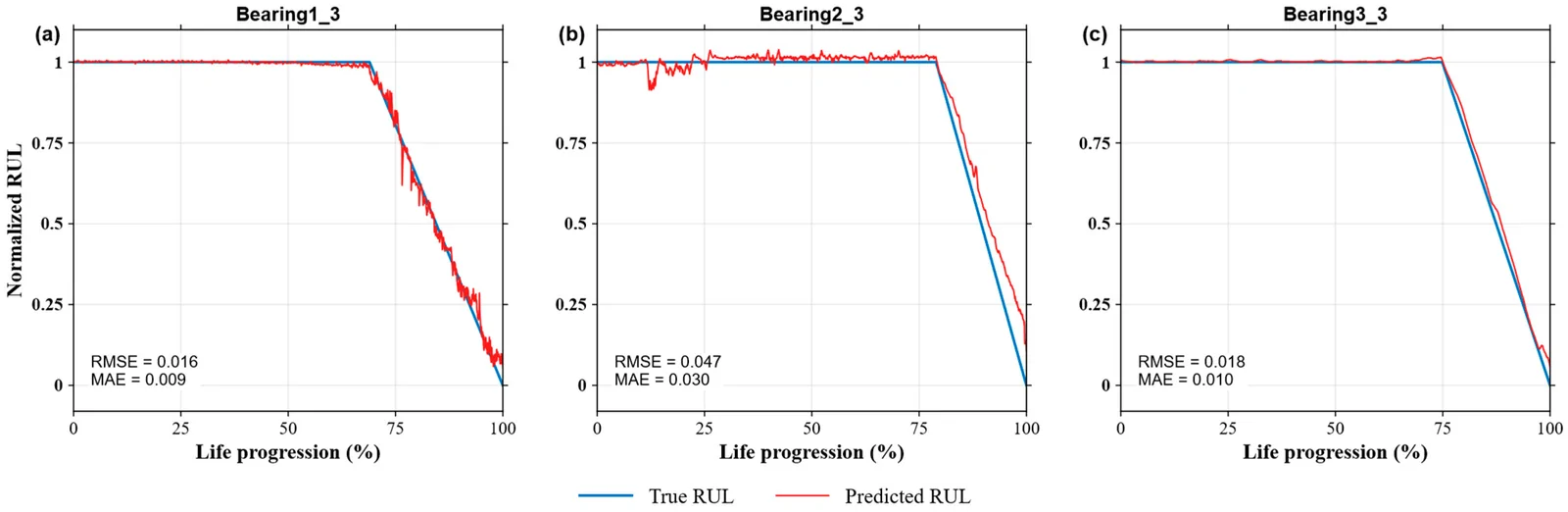
Normalized RUL predictions of SCDB-LSTM for test bearings under the three operating conditions: (**a**) Bearing1_3 under Condition 1; (**b**) Bearing2_3 under Condition 2; and (**c**) Bearing3_3 under Condition 3.

To examine temporal prediction behavior beyond the aggregate metrics, Figure 7 presents the normalized RUL trajectories produced by SCDB-LSTM for one test bearing from each operating condition. Bearing1_3, Bearing2_3 and Bearing3_3 were selected because they occupy the same predefined position as the first test bearing within their respective condition-specific subsets, rather than because of their prediction accuracy.

Figure 7 juxtaposes the prediction and reference target for one test bearing from each operating condition. These examples visualize tracking behavior but do not replace the complete bearing-level evaluation or establish run-to-run stability.

The progressive and abrupt degradation patterns discussed in Section 3.2 provide additional context for interpreting the Condition 1 results. Bearings with an extended progressive-degradation interval provide a longer sequence over which the stage-aware temporal relationship can be learned and evaluated. By contrast, abruptly degrading bearings contain fewer post-FPT observations and may therefore produce larger prediction deviations near the failure endpoint. The bearing-specific FPT and causal stage coefficient allow SCDB-LSTM to accommodate these differences without imposing an identical transition position on every bearing.

Overall, the results demonstrate the performance of SCDB-LSTM when the experimental evaluation is extended from Condition 1 to three operating conditions with different rotational speeds and radial loads. Because a separate model is trained and tested within each condition, these experiments assess the repeatability of the proposed framework under multiple operating conditions but do not constitute cross-condition transfer or unseen-condition generalization. The contributions of the principal framework components are examined further through the Condition 1 ablation study in Section 3.4.

### 3.4. Ablation and Sensitivity Analysis

Ablation experiments were conducted under Condition 1 to evaluate the contributions of the principal framework components. Bearing1_1 and Bearing1_2 were used for model development, while Bearing1_3–Bearing1_5 were used for testing. All variants followed the same preprocessing, training, and five-random-seed protocol. MAE and RMSE were calculated separately for each test bearing and then averaged across the three test bearings.

The complete framework was compared with five variants. Fixed-fusion HI removes the mechanism-informed gate and recomputes the downstream FPT and stage coefficient from the resulting HI. Direct concatenation replaces the two branch-specific mappings with a common mapping of the concatenated inputs while retaining the shared LSTM. The full-life linear-target variant replaces the FPT-aligned training target with a conventional full-life target. The remaining variants remove stage conditioning or replace the bearing-stage-weighted Huber objective with unweighted MSE.

Table 8 reports MAE and RMSE values of 0.009 and 0.016 for the complete framework. Each listed ablation has larger errors in this table. Fixed fusion changes the HI and its downstream transition and stage quantities, so its difference measures a pipeline-level effect rather than isolated rejection of transient shocks. The optimization ablation changes both balancing weights and loss type and therefore does not separate their individual contributions. The full-life linear-target variant changes the supervision definition; a comparison is interpretable only if predictions are evaluated against a clearly specified common target, and does not establish superiority for physical remaining-time prediction.

A compact sensitivity analysis was further performed by perturbing the detected FPT and three representative parameters around their reported settings. Only one factor was changed at a time, while the remaining settings and the evaluation target were kept fixed.

The reported values in Table 9 indicate substantial sensitivity to the evaluated perturbations. Based on the displayed means, shifting the FPT by −10% and +10% of the sequence length increases the MAE from 0.009 to 0.158 and 0.187, respectively, while the corresponding RMSE increases from 0.016 to 0.184 and 0.214. Among the evaluated settings, the FPT perturbations produce the largest prediction errors. Changes in the gate threshold, Level threshold and stage-smoothing coefficient also increase the prediction errors relative to the reference configuration. These results indicate that transition calibration and representative parameter selection remain important to the current framework, with the FPT location having a particularly pronounced influence on downstream prediction performance.

For implementation, the two vibration channels must remain synchronized, and the fault-frequency bands require an appropriate speed and bearing-geometry specification. Feature extraction and trailing evidence windows can be updated record by record after calibration. For a fixed sequence length T, LSTM input dimension d and hidden size h, the recurrent work per prediction scales as O(T h(d + h)); exact timing also includes signal processing and the input mappings. No measured runtime or memory benchmark is reported here, so compatibility with the 10 s acquisition interval is a deployment requirement rather than a demonstrated real-time result. A prospective replay test should confirm both timing and the absence of future-data dependencies.

## 4. Conclusions

This study develops a sequential bearing-prognostic framework that links mechanism-informed health representation, causal transition confirmation and stage-conditioned prediction. MIG-HI regulates impulsive evidence using fault-frequency and cross-channel support. CETI combines level, trend and persistence evidence to establish a formal FPT, which informs offline target construction and the inference-time stage coefficient. SCDB-LSTM combines branch-specific mappings, a shared unidirectional LSTM and stage-conditioned regression. The Condition 1 HI comparison shows the highest trendability for MIG-HI but also its lowest monotonicity among the evaluated indicators. The tabulated condition-specific prediction results favor the proposed method under Conditions 1 and 3 and DTA-MLP under Condition 2. The ablation and sensitivity analyses must be interpreted within their stated protocols and do not establish universal superiority or insensitivity to transition calibration.

The evaluation described in this manuscript uses a selected subset of 13 bearings from three operating conditions, with separate model development within each condition. It therefore does not demonstrate cross-condition transfer, generalization to unseen bearing types, or deployment under variable industrial loads and speeds. Five-seed summaries describe training variability but do not by themselves establish statistical significance across independent bearings. Controlled disturbance tests, alternative transition-detector comparisons, complete baseline specifications, and reliable uncertainty estimates remain necessary for broader claims. The normalized target is not a direct estimate of remaining operating time, and the initial calibration interval delays the start of causal monitoring. Future work should address these limitations and evaluate physical-time predictions on independent data.

## Figures and Tables

**Table 1:** The composition and functional roles of the degradation evidence used in MIG-HI.

Evidence	Representative Descriptors	Construction	Functional Role
$D_{t}$ structural deviation	Statistical and distributional descriptors; spectral-shape descriptors	Two-sided activation and mean aggregation	Describes general departure from the healthy reference
$E_{t}$ energy-related degradation	RMS, amplitude-related descriptors, envelope energy and frequency-band energy	Positive activation and upper-fraction aggregation	Characterizes energy redistribution and growth
$I_{t}$ impulsiveness	Kurtosis-, peak-, crest- and impulse-related descriptors	Positive activation and upper-fraction aggregation	Captures localized impulsive responses; its final contribution is regulated by the gate
$M_{t}$ fault mechanism support	Envelope-spectrum energy around BPFO, BPFI, BSF, FTF and their harmonics	Positive activation and upper-fraction aggregation	Represents fault-related spectral concentration and provides mechanism support for gating
$C_{t}$ cross-channel consistency	Absolute inter-channel correlation; RMS, peak-to-peak, spectral-energy and envelope-energy ratio symmetry	Bounded consistency mapping and upper-fraction aggregation	Provides corroborative evidence of coordinated horizontal–vertical responses

**Table 2:** Causal evidence used for degradation-transition identification in CETI.

Evidence	Quantity	Decision Criterion	Functional Role
Level	$L_{t}$	$L_{t}\geq \theta_{L}$	Detects a significant instantaneous departure from the healthy HI distribution
Trend	$T_{t},\beta_{t}$	$T_{t}\geq \theta_{T}\;\;\mathrm{and}\;\;\beta_{t}>0$	Confirms that the recent HI evolution is directed toward degradation
Persistence	$P_{t},n_{t}^{con}$	$P_{t}\geq \eta_{P\;\;}\mathrm{and}\;\;n_{t}^{con}\geq N_{P}$	Confirms that weaker abnormal deviations have become temporally sustained

**Table 3:** The operating conditions and the selected learning and test bearings used in this study.

Condition	Rotational Speed (r/min)	Radial Load (N)	Learning Bearings	Test Bearings
Condition 1	1800	4000	Bearing1_1, Bearing1_2	Bearing1_3–Bearing1_5
Condition 2	1650	4200	Bearing2_1, Bearing2_2	Bearing2_3–Bearing2_5
Condition 3	1500	5000	Bearing3_1, Bearing3_2	Bearing3_3

**Table 4:** The main implementation settings used in the PHM 2012 experiments.

Setting	Value	Setting	Value
Sampling configuration	25.6 kHz; 2560 points/channel; interval = 10 s	SCDB-LSTM sequence length (*T*)	30
MIG-HI calibration prefix (*N_c_*)	200	HI trend window	10
MIG-HI reference window (*N_r_*)	100	Stage-smoothing coefficient/scale quantile (*α_c_*/*q_c_*)	0.10/0.75
Reference-slope quantile (*q_s_*)	0.80	LSTM hidden/head units	64/32
Activation threshold/width (*θ_a_*/*w_a_*)	1/3	HI/feature mapping units	16/48
Gate minimum/threshold/temperature ($g_{min}$/*θ_g_*/*T_g_*)	0.15/1/0.20	Maximum epochs/batch size	100/128
Fusion weights (*w_D_*/*w_E_*/*w_M_*/*w_I_*)	0.20/0.30/0.25/0.25	Learning rate/early-stopping patience	1 × 10^−3^/15
MIG-HI EWMA coefficient (*α_H_*)	0.10	Number of random seeds	5
CETI Level threshold (*θ_L_*)	3	CETI Persistence fraction/consecutive records (*η_P_*/*N_P_*)	0.75/4
CETI Trend window/threshold (*W_T_*/*θ_T_*)	20/2	Training loss/Huber threshold (*δ*)	Bearing-stage weighted Huber/0.10
CETI weak-Level threshold/Persistence window (*θ_P_*/*W_P_*)	1/12		

**Table 5:** Quantitative comparison of different health indicators under Condition 1.

Health Indicator	Monotonicity	Trendability	Prognosability
RMS-HI	0.4341	0.2169	0.8897
Kurtosis-HI	0.1941	0.0195	0.6809
PCA-HI	0.3889	0.0342	0.9946
MIG-HI	0.1575	0.4967	0.8560

**Table 6:** Formal FPTs identified by CETI for the five bearings under Condition 1.

Bearing	Formal FPT	Relative FPT Position (%)	Remaining Records After FPT	Degradation Pattern
Bearing1_1	1200	42.791	1604	Progressive
Bearing1_2	810	92.989	62	Abrupt
Bearing1_3	800	33.656	1576	Progressive
Bearing1_4	1080	75.613	349	Progressive
Bearing1_5	2400	97.441	64	Abrupt

**Table 7:** Normalized RUL prediction performance under the three operating conditions. The results are reported as the mean ± standard deviation over five random seeds.

Method	Condition 1 MAE	Condition 1 RMSE	Condition 2 MAE	Condition 2 RMSE	Condition 3 MAE	Condition 3 RMSE	Overall MAE	Overall RMSE
LSTM	0.040 ± 0.02	0.067 ± 0.02	0.105 ± 0.02	0.128 ± 0.02	0.029 ± 0.02	0.047 ± 0.02	0.066 ± 0.02	0.090 ± 0.02
TCN	0.023 ± 0.02	0.039 ± 0.02	0.086 ± 0.02	0.118 ± 0.02	0.034 ± 0.02	0.062 ± 0.02	0.052 ± 0.02	0.076 ± 0.02
MDSCT [24]	0.016 ± 0.02	0.026 ± 0.02	0.072 ± 0.02	0.093 ± 0.02	0.021 ± 0.02	0.044 ± 0.02	0.041 ± 0.02	0.057 ± 0.02
DTA-MLP [25]	0.013 ± 0.02	0.031 ± 0.02	0.028 ± 0.02	0.042 ± 0.02	0.018 ± 0.02	0.026 ± 0.02	0.020 ± 0.02	0.035 ± 0.02
Proposed	0.009 ± 0.02	0.016 ± 0.02	0.030 ± 0.02	0.047 ± 0.02	0.010 ± 0.02	0.018 ± 0.02	0.018 ± 0.02	0.030 ± 0.02

**Table 8:** Component-ablation results under Condition 1. The results are averaged across Bearing1_3–Bearing1_5 and reported as the mean ± standard deviation over five random seeds.

Variant	Main Modification	Overall MAE	Overall RMSE
Full framework	MIG-HI + CETI + SCDB-LSTM	0.009 ± 0.002	0.016 ± 0.003
Fixed-fusion HI	Remove mechanism-informed gating	0.054 ± 0.002	0.083 ± 0.003
Direct concatenation	Replace the branch-specific mappings with a common input mapping	0.070 ± 0.002	0.132 ± 0.003
Full-life linear target	Replace FPT-aligned supervision	0.129 ± 0.002	0.171 ± 0.003
w/o stage conditioning	Remove the causal stage coefficient from the regression head	0.112 ± 0.002	0.150 ± 0.003
w/o balanced robust optimization	Replace weighted Huber with unweighted MSE	0.109 ± 0.002	0.146 ± 0.003

**Table 9:** Sensitivity to FPT perturbations and representative parameter settings under Condition 1.

Setting	Perturbation	Overall MAE	Overall RMSE
Reference configuration	-	0.009 ± 0.002	0.016 ± 0.003
FPT position	−10% of sequence length	0.158 ± 0.002	0.184 ± 0.003
	+10% of sequence length	0.187 ± 0.002	0.214 ± 0.003
Gate threshold ($\theta_{g}$)	0.90	0.137 ± 0.002	0.157 ± 0.003
	1.05	0.123 ± 0.002	0.144 ± 0.003
Level threshold ($\theta_{L}$)	2.85	0.097 ± 0.002	0.126 ± 0.003
	3.15	0.129 ± 0.002	0.147 ± 0.003
Stage-smoothing coefficient ($\alpha_{c}$)	0.090	0.086 ± 0.002	0.111 ± 0.003
	0.110	0.108 ± 0.002	0.123 ± 0.003

## Data Availability

Publicly available datasets were analyzed in this study. The IEEE PHM 2012 Data Challenge (PRONOSTIA) bearing dataset is publicly available at https://github.com/Lucky-Loek/ieee-phm-2012-data-challenge-dataset (accessed on 1 October 2026).

## Abbreviations

The following abbreviations are used in this manuscript:

RUL	Remaining Useful Life
MIG-HI	Mechanism-Informed Gated Health Indicator
CETI	Causal Evidence-Driven Transition Identification
SCDB-LSTM	Stage-Conditioned Dual-Branch Long Short-Term Memory Network
FPT	First Prediction Time
HI	Health Indicator
MAE	Mean Absolute Error
RMSE	Root Mean Square Error
LSTM	Long Short-Term Memory
MAD	Median Absolute Deviation
BPFO	Ball-Pass Frequency of the Outer Race
BPFI	Ball-Pass Frequency of the Inner Race
BSF	Ball Spin Frequency
FTF	Fundamental Train Frequency
RMS	Root Mean Square
PCA	Principal Component Analysis
CPU	Central Processing Unit
GPU	Graphics Processing Unit
EWMA	Exponentially Weighted Moving Average
RMS-HI	Root-Mean-Square-Based Health Indicator
PCA-HI	Principal-Component-Analysis-Based Health Indicator
GRU	Gated Recurrent Unit
TCN	Temporal Convolutional Network
CNN	Convolutional Neural Network
MSE	Mean Squared Error
